# Geographic Examination of COVID-19 Test Percent Positivity and Proportional Change in Cancer Screening Volume, National Breast and Cervical Cancer Early Detection Program

**DOI:** 10.5888/pcd19.220111

**Published:** 2022-09-15

**Authors:** Yamisha Bermudez, Lia C. Scott, Michele Beckman, Amy DeGroff, Kristy Kenney, Juzhong Sun, Tanner Rockwell, William Helsel, William Kammerer, Amy Sheu, Jacqueline Miller, Lisa C. Richardson

**Affiliations:** 1 Totally Joined for Achieving Collaborative Techniques, Atlanta, Georgia; 2 Georgia State University, Atlanta, Georgia; 3 Division of Cancer Prevention and Control, Centers for Disease Control and Prevention, Atlanta, Georgia; 4 Information Management Services, Inc, Calverton, Maryland

## Abstract

**Introduction:**

In 2020, the COVID-19 pandemic led to significant declines in cancer screening, including among women served by the National Breast and Cervical Cancer Early Detection Program (NBCCEDP). This study examined the spatial association between state-based COVID-19 test percent positivity and proportional change in NBCCEDP screening volume.

**Methods:**

Using the COVID-19 Diagnostic Laboratory Testing dataset, we calculated state-based monthly COVID-19 test percent positivity from July through December 2020 and categorized rates into low, medium, and high groups. We used data from 48 NBCCEDP state awardees to calculate the state-based monthly proportional change in screening volume and compared data for July–December 2020 with the previous 5-year average for those months. We categorized changes in screening volume into large decrease, medium decrease, and minimal change and created maps of the associations between variable subgroups by using bivariate mapping in QGIS.

**Results:**

Bivariate relationships between COVID-19 test percent positivity and proportional change in cancer screening volume varied over time and geography. In 5 of 6 months, 4 states had high COVID-19 test percent positivity and minimal change in breast *or* cervical cancer screening volume; 2 states had high COVID-19 test percent positivity and minimal change in breast *and* cervical cancer screening volume.

**Conclusion:**

Some states maintained pre–COVID-19 screening volumes despite high COVID-19 test percent positivity. Follow-up research will be conducted to determine how these states differ from those with consistent decreases in screening volume and identify factors that may have contributed to differences. This information could be useful for planning to maximize NBCCEDP awardees’ ability to maintain screening volume during future public health emergencies.

**Summary**

**What is already known on this topic?**

In 2020, the COVID-19 pandemic led to significant declines in cancer screening, including among women served by the National Breast and Cervical Cancer Early Detection Program.

**What is added by this report?**

The bivariate relationship between COVID-19 test percent positivity and proportional change in cancer screening volume varied across states and over time. We identified states that consistently maintained pre–COVID-19 breast and/or cervical cancer screening volumes despite consistently high COVID-19 test percent positivity.

**What are the implications for public health practice?**

Identified states may have strategies that can help other states maintain levels of preventive health services during future public health emergencies.

## Introduction

Cancer screening is an effective strategy to reduce breast and cervical cancer morbidity and mortality (1–4). The National Breast and Cervical Cancer Early Detection Program (NBCCEDP) provides breast and cervical cancer screening, diagnostic testing, and cancer treatment referral services to low-income women who are underinsured and uninsured. Funded by the Centers for Disease Control and Prevention (CDC), NBCCEDP awardees include 50 states, the District of Columbia, and 19 tribal and territorial organizations.

The COVID-19 pandemic disrupted cancer control activities in the US (5). In April 2020, breast and cervical cancer screening volume in the NBCCEDP declined by 87% and 84%, respectively, compared with the previous 5-year average for that month (6). The extent of this decline varied across the US Department of Health and Human Services geographic regions: Region 2, New York, experienced the largest declines in both breast and cervical cancer screening volume, and Region 7, Kansas City, had the smallest declines (6). Place-based surges in COVID-19 infections and related declines in screening volume may lead to delayed cancer diagnosis and treatment and, therefore, geographic disparities in cancer mortality (7).

This study examined the spatial distribution of the association between COVID-19 test percent positivity and proportional changes in NBCCEDP cancer screening volume, by state, during a 6-month period, July through December 2020. We used bivariate maps to identify states with 1) consistently high COVID-19 test percent positivity and large proportional declines in screening volume, 2) consistently high COVID-19 test percent positivity and minimal proportional change in cancer screening volume, and 3) a downward trajectory in COVID-19 test percent positivity and a gradual recovery in cancer screening volume, concurrently, over time. States with high COVID-19 test percent positivity and minimal proportional change in cancer screening volume may be further examined to identify potential strategies to maximize NBCCEDP awardees’ ability to maintain screening volume during future public health emergencies.

## Methods

For this descriptive study, we used the COVID-19 Diagnostic Laboratory Testing dataset provided by the US Department of Health and Human Services to create a COVID-19 test percent positivity measure (8). This time-series dataset includes geocoded polymerase chain reaction (PCR) testing results reported to state and jurisdictional health departments from more than 1,000 laboratories and testing locations in the US. These data represent diagnostic specimens tested, not individuals (8). To calculate the test percent positivity by state for each month of the 6-month study period, we divided the cumulative number of positive COVID-19 tests by the total number of tests performed per month and multiplied that quotient by 100 (9).

We also used NBCCEDP program data, the minimal data elements (MDEs), that are collected for all women receiving services through the NBCCEDP awardees and reported to CDC (10). Each MDE record describes a screening cycle that starts with receipt of a screening test and follows women through diagnostic follow-up of abnormal test results and, if diagnosed with cancer, a treatment referral. Each encounter of a given cycle with a woman is included in the MDE record; therefore, multiple tests per woman may be included in the MDE record. MDE variables include patient demographic characteristics, screening date, test result, final diagnosis (cancer or no cancer), the date of the final diagnosis, and treatment initiation date, if indicated (11). The MDEs have Office of Management and Budget (no. 0920–0571) and CDC human subjects approvals.

We aggregated MDE records by state and assessed the state-level proportional change in breast cancer screening tests (mammograms) and cervical cancer screening tests (Papanicolaou tests and/or human papillomavirus tests) compared with the previous 5-year average (2015–2019), by month, for the 6-month study period, July–December 2020. To calculate proportional change in cancer screening tests by state for each month of the 6-month study period, we divided the difference in screening tests volume between 2020 and the previous 5-year average (2015–2019) for each state by the previous 5-year average for each state and multiplied the quotient by 100. Data from 48 of the 70 NBCCEDP awardees (48 states) were included in the analysis. We excluded awardees with either insufficient spatial data (all tribal organizations and territories) or missing 2020 data for breast and/or cervical cancer screening (District of Columbia, Massachusetts, and North Carolina).

### Data analysis

We used QGIS version 3.16.9 (Open Source Geospatial Foundation) to create a series of bivariate maps to display the associations between COVID-19 test percent positivity and 1) proportional change in NBCCEDP breast cancer screening volume and 2) proportional change in NBCCEDP cervical cancer screening volume. Associations were mapped for each month of the study period, July–December 2020, for a total of 12 maps. We used tertiles to determine bivariate categories for proportional change in screening volume. This method sorted the state-level proportional change in breast and cervical cancer screening volume from lowest to highest value and divided the distribution of the data into 3 equal parts. Proportional changes in breast and cervical cancer screening volume values in the first tertile were considered a large decrease (< −20%), values in the second tertile a medium decrease (−20% to −5%), and values in the third tertile a minimal change (> −5%). This third tertile included states that had a small proportional decrease or increase.

To determine bivariate categories for COVID-19 test percent positivity, we used CDC’s thresholds for community transmission levels of SARS-CoV-2, which account for both the number of new cases and the test percent positivity in the past 7 days. With these thresholds, transmission level is categorized as low (<5.00% positivity), moderate (5.00%–7.99% positivity), substantial (8.00%–9.99% positivity), or high (≥10.00%) (12). For this analysis, we combined substantial and high transmission categories into a single category, high transmission (≥8.00%).

## Results

During July–December 2020, a total of 22,465,958,918 COVID-19 tests were performed in the 48 states included in the study, with 1,885,013,432 confirmed positive results. The 6-month average COVID-19 test percent positivity across these states was 8.39%. The monthly test percent positivity gradually declined from July (8.25%) through October (7.31%) and then sharply increased through December (9.11%).

During July–December 2020, a total of 93,166 breast cancer screening tests and 84,721 cervical cancer screening tests were performed by the NBCCEDP state awardees included in the study. Compared with the previous 5-year average for the same 6 months, 21,597 fewer breast cancer screening tests and 21,478 fewer cervical cancer screening tests were performed. We found an average decline of 73.3 breast cancer screening tests and 71.7 cervical cancer screening tests per month in each state. Average proportional declines in breast and cervical cancer screening volume across states more than doubled from September to November 2020 when compared with the previous 5-year average, then improved in December (Table). However, we observed the largest proportional declines in July (−22.9% breast, −24.9% cervical), August (−25.2% breast, −25.5% cervical), and November (−22.7% breast, −24.9% cervical).

**Table:** Monthly Breast and Cervical Cancer Screening Volume for July–December 2020 Compared With Previous 5-Year Average 2015-2019^a^, National Breast and Cervical Cancer Early Detection Program

Item	July	August	September	October	November	December
**Breast cancer screening volume**						
2020	13,523	14,525	16,080	18,936	15,347	14,755
5-year average (2015–2019)	17,529	19,467	18,087	22,332	19,832	17,515
Percentage decrease	−22.9	−25.2	−11.2	−15.3	−22.7	−16.0
**Cervical cancer screening volume**						
2020	12,354	13,742	15,990	17,453	13,359	11,823
5-year average (2015–2019)	16,432	18,411	17,798	20,980	17,791	14,784
Percentage decrease	−24.9	−25.5	−10.2	−16.9	−24.9	−20.1

a Breast and cervical cancer screening volume is based on minimal data elements submitted in April 2021. Screening data exclude North Carolina, Massachusetts, and the District of Columbia because of a lack of 2020 screening data.

### Breast cancer screening volume and COVID-19 test percent positivity

**Figure 1:**
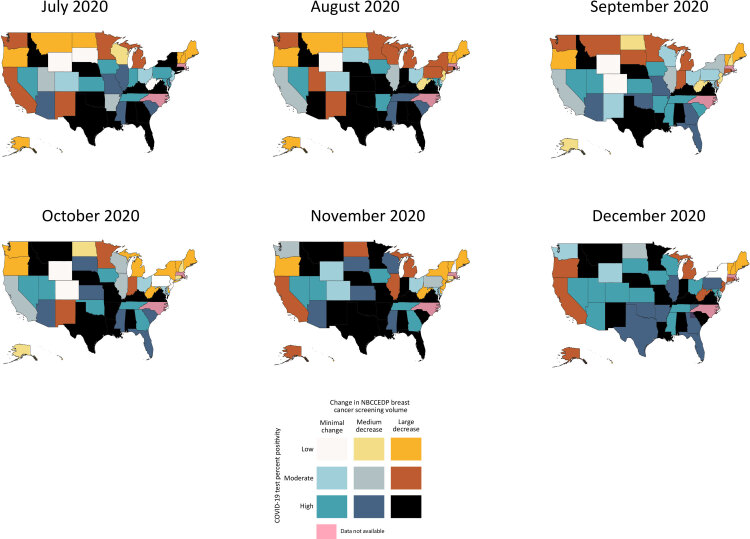
Bivariate visualization of the association between state-level proportional change in NBCCEDP breast cancer screening volume and COVID-19 test percent positivity for each month from July through December 2020. Breast cancer screening volume was based on NBCCEDP minimal data elements submitted in April 2021. Data for the District of Columbia, Massachusetts, and North Carolina and are not displayed because 2020 breast cancer screening data were missing. The change in cancer screening volume was calculated as the difference between the volume during July–December 2020 and the previous 5-year average for those months. Abbreviation: NBCCEDP, National Breast and Cervical Cancer Early Detection Program.

Seven states had a bivariate relationship characterized by consistently high COVID-19 test percent positivity and large proportional decreases in breast cancer screening volume (Figure 1). For at least 5 of 6 months, Alabama, Idaho, Kentucky, Louisiana, Nebraska, Texas, and Virginia demonstrated this relationship. In contrast, Iowa, Nevada, and Tennessee experienced high COVID-19 test percent positivity and minimal proportional change in breast cancer screening volume in at least 5 of 6 months (Figure 1).

Among states with high COVID-19 test percent positivity, more states concurrently had a large decrease in breast cancer screening volume than a minimal proportional change in 4 of 6 months (Figure 1). For example, among states with high COVID-19 test percent positivity in November, 16 states concurrently had large proportional decreases in breast cancer screening volume and only 5 states had minimal proportional change. However, in September 2020, this relationship was reversed: 8 states had minimal proportional change and 7 states had a large proportional decrease in breast cancer screening volume while concurrently having high COVID-19 test percent positivity. The same number of states (n = 12) showed these 2 bivariate relationships in December 2020.

No state simultaneously demonstrated a downward trajectory in COVID-19 test percent positivity and a gradual recovery in proportional change in breast cancer screening volume over time (Figure 1). Most states with high COVID-19 test percent positivity and large proportional decreases in breast cancer screening volume maintained that relationship through December 2020. Among states with high COVID-19 test percent positivity, few moved back and forth between large and medium proportional decreases in breast cancer screening volume. Georgia showed a gradual recovery in proportional change in breast cancer screening volume while experiencing consistently high COVID-19 test percent positivity. While maintaining high COVID-19 test percent positivity, the proportional change in Georgia’s breast cancer screening volume shifted from a large decrease in July and August, to a medium decrease in September, to a minimal proportional change in October and November. However, in December 2020, the relationship went back to high COVID-19 test percent positivity and medium proportional declines in breast cancer screening volume.

### Cervical cancer screening volume and COVID-19 test percent positivity

**Figure 2:**
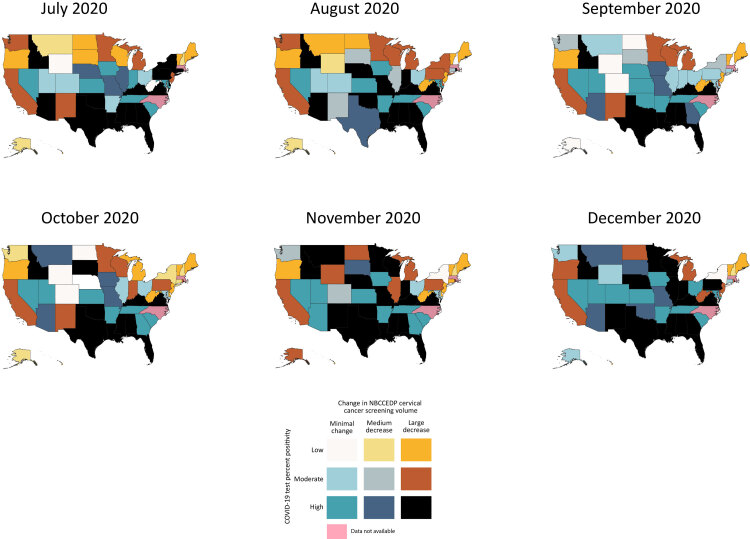
Bivariate visualization of the association between state-level proportional change in NBCCEDP cervical cancer screening volume and COVID-19 test percent positivity for each month from July through December 2020. Cervical cancer screening volume was based on NBCCEDP minimal data elements submitted in April 2021. Data for the District of Columbia, Massachusetts, and North Carolina and are not displayed because 2020 cervical cancer screening data were missing. The change in cancer screening volume was calculated as the difference between the volume during July–December 2020 and the previous 5-year average for those months. Abbreviation: NBCCEDP, National Breast and Cervical Cancer Early Detection Program.

Eight states consistently experienced high COVID-19 test percent positivity and large decreases in cervical cancer screening volume (Figure 2). In at least 5 of 6 months, Alabama, Florida, Idaho, Kentucky, Louisiana, Mississippi, Virginia, and Texas demonstrated this negative association. In contrast, Arkansas, Kansas, Nevada, South Carolina, and Tennessee showed high COVID-19 test percent positivity and minimal proportional change in cervical cancer screening in at least 5 of 6 months.

Among states with high COVID-19 test percent positivity, more states concurrently had a large proportional decrease than a minimal proportional change in cervical cancer screening volume in 5 of 6 months (Figure 2). For example, among states with high COVID-19 test percent positivity in July, 16 states concurrently had large proportional decreases in cervical cancer screening volume and only 9 states had minimal proportional change. However, only 8 states showed these 2 relationships in September. During the 6-month period, we observed the greatest number (n = 17) of states with high COVID-19 test percent positivity and large proportional decreases in cervical cancer screening volume in July. We found the greatest number (n = 10) of states with high COVID-19 test percent positivity and minimal proportional change in cervical cancer screening volume in December.

No states simultaneously demonstrated a downward trajectory in COVID-19 test percent positivity and a gradual recovery in proportional change in cervical cancer screening volume over time. Similar to the patterns for breast cancer, most states with high COVID-19 test percent positivity and large proportional decreases in cervical cancer screening volume maintained that bivariate relationship through December (Figure 2).

### Co-location of bivariate relationships

Six states had high COVID-19 test percent positivity and large proportional decreases in screening volume for both breast and cervical cancer in at least 5 of 6 months: Alabama, Idaho, Kentucky, Louisiana, Texas, and Virginia. In contrast, Nevada and Tennessee demonstrated a relationship characterized by high COVID-19 test percent positivity and minimal proportional change in screening volume for both cancers in at least 5 of 6 months. Finally, 1 state, Oregon, had low COVID-19 test percent positivity and large proportional decreases in screening volume for both breast and cervical cancer for the first 5 months, July through November. 

## Discussion

We conducted the first study to our knowledge that examines the geospatial relationship between breast and cervical cancer screening in a large population of low-income women who are uninsured or underinsured, and state-level COVID-19 test percent positivity. Although several studies reported significant declines in cancer screening early in the pandemic (5,6,13), our study aimed to visually describe the relationship between state-level COVID-19 test percent positivity and proportional change in breast and cervical cancer screening volume in the NBCCEDP and how the relationship differed by geography in each month from July through December 2020.

We found substantial variations in COVID-19 test percent positivity over time and geography. Overall, these variations aligned with the temporal and spatial distributions of proportional change in NBCCEDP cancer screening volume, with states mostly showing large proportional declines in cancer screening volume when and where COVID-19 test percent positivity was highest. When we examined bivariate associations by state, the number of states with high COVID-19 test percent positivity and large proportional decreases in cancer screening volume was larger than the number of states with high COVID-19 test percent positivity and minimal proportional change in 4 of 6 months for breast cancer and 5 of 6 months for cervical cancer. However, the same number of states had these relationships for cervical cancer in September 2020 (8 states had high-COVID-19/large decrease and 8 states had high-COVID-19/minimal change) and for breast cancer in December 2020 (12 states for both bivariate relationships). Further research may identify factors that help to explain why we found an equal number of states with high-COVID-19/large decrease and high-COVID-19/minimal change relationships in September and December while we found differences in the number of states with these relationships during other months. Findings from this additional research could inform the development of time-specific strategies that could help states maintain receipt of preventive health services during highly affected points in future public health emergencies. We also found that by November 2020, only 1 state, New York, showed a relationship characterized by low COVID-19 test percent positivity and minimal proportional change in cancer screening volume (the best-case scenario) for breast and cervical cancer. Very few states showed this relationship before November, suggesting that during our study period, the COVID-19 pandemic may have caused an overall lack of capacity to deliver care as health care workers were being pulled to work on the pandemic (14).

Although COVID-19 test percent positivity and proportional change in cancer screening volume among women in the NBCCEDP appear to be influenced by time, both also appear to be influenced by an element of geography. One study found that Region 2, New York, experienced the greatest decline in cancer screening volume in April 2020, and Region 7, Kansas City, experienced the smallest decline (6). However, when we examined the bivariate relationships between COVID-19 test percent positivity and proportional change in NBCCEDP cancer screening volume, we identified other geographic areas most affected during July–December 2020. We found that 6 states showed high COVID-19 test percent positivity and a large proportional decrease in screening volume for both breast and cervical cancer in at least 5 of 6 months during the study period: Alabama, Idaho, Kentucky, Louisiana, Texas, and Virginia. Apart from Idaho, all states showing this relationship for breast and cervical cancer are in the Southern region. Some factors that may be associated with the findings for the Southern region include limited access to health care, high poverty rates, and a large African American population (15–19). Over the past 10 years, 138 rural hospitals in the US closed; more than 60% of them were in the South (15). Also, the South has historically had higher poverty rates than other regions. Nearly 84% of persistent-poverty counties (≥20% of their populations living in poverty based on 1980, 1990, and 2000 decennial censuses and 2007–2011 American Community Survey 5-year estimates) were in the South (16). People of low socioeconomic status (SES) are more likely than people with higher SES to have essential jobs, which may prevent them from self-isolating, thereby increasing their risk of COVID-19 exposure (17). Finally, more than half of African American people in the US, who are contracting COVID-19 at a disproportionately higher rate and experiencing worse COVID-19–related outcomes compared with White people, live in the South (18,19). Together these factors are associated with higher risk of COVID-19 exposure in the South than in other regions of the US, and our study results suggest an association between places with higher COVID-19 test percent positivity and larger declines in cancer screening volume. However, in other states, such as Nevada and Tennessee, we found high COVID-19 test percent positivity and minimal proportional change in cancer screening volume for both breast and cervical cancer in at least 5 of 6 months during the study period. Despite consistently high COVID-19 test percent positivity in these states, they maintained pre–COVID-19 breast and cervical cancer screening volumes. Similarly, Georgia’s bivariate relationships over time also stood out: this state gradually moved toward pre–COVID-19 screening volumes despite consistently high COVID-19 positivity rates. We plan to conduct follow-up research to determine what actions taken by these NBCCEDP state awardees and their clinics may have contributed to the consistent maintenance of pre–COVID-19 screening volume or gradual improvement despite high COVID-19 test percent positivity and how these actions differed from those taken by states that consistently showed decreases in screening volume.

### Limitations

Our study has several limitations. First, consolidating CDC’s COVID-19 thresholds for determining community transmission levels of SARS-CoV-2 in a different way would likely have resulted in different bivariate patterns in states and over time. Because a systematic review of the literature on COVID-19 spatial analysis indicated no examples of bivariate mapping visualization methods (20), we had no existing categorization approaches to guide our novel analysis. Second, using a different COVID-19 indicator (eg, COVID-19 case rate, case count, hospitalization rate) might have resulted in different patterns in states and over time. We used COVID-19 test percent positivity because the data for this indicator were more complete than other potential indicators across states and during the study period. Third, COVID-19 test percent positivity data represent diagnostic specimens tested. Therefore, COVID-19 test percent positivity depends on the number of tests performed, not the number of people, which could lead to underestimates of COVID-19 test percent positivity. Fourth, although we offered a few likely explanations for some of the bivariate patterns observed, the factors contributing to each of these patterns are unknown and most likely multifactorial. The bivariate relationship between COVID-19 test percent positivity and proportional change in NBCCEDP cancer screening volume observed in each state may have been affected by a combination of factors existing at the county, community, health system, patient, health care provider, or policy levels.

### Conclusion

Our geospatial analysis demonstrated geographic differences in the proportional change in cancer screening volume in the NBCCEDP and COVID-19 test percent positivity across the US in the second half of 2020, which allows us to better understand the impact of COVID-19 on the program. Place-based surges in COVID-19 infections and related declines in screening volume may lead to delayed cancer diagnosis and treatment and, therefore, geographic disparities in cancer mortality (7). Additionally, existing disparities in breast and cervical cancer incidence and mortality could be exacerbated, given the disproportionate impact of the pandemic on racial and ethnic minority populations (21). Our study identified states that maintained pre–COVID-19 breast and/or cervical cancer screening volumes despite high COVID-19 test percent positivity during July–December 2020. Follow-up research on these states may inform strategies that can help other states maintain delivery of preventive health services during future public health emergencies.
